# Gender Differences in Geriatric Depressive Symptoms in Rural China: The Role of Physical Housing Environments and Living Arrangements

**DOI:** 10.3390/ijerph16050774

**Published:** 2019-03-04

**Authors:** Mingwang Fang, Jinfeng Chen, Ling Guo, Xiao Ma

**Affiliations:** 1 Department of Health-Related Social and Behavioral Science, West China School of Public Health, Sichuan University, Chengdu 610041, China; fangmw1991@163.com (M.F.); chenjf950711@163.com (J.C.); 2 Department of Health management, Chongqing Nursing Vocational College, Chongqing 402763, China; linlingguo92@163.com

**Keywords:** gender difference, geriatric depressive symptoms, physical housing environments, living arrangements, rural China

## Abstract

Physical housing environment and living arrangements are significant determinants of health, particularly in developing countries, although results are mixed. We conducted this study to examine the gender differences in geriatric depressive symptoms in rural China, and further explored the influence of housing environments and living arrangements on depressive symptoms. The data used for this study were from the third wave of the nationally representative China Health and Retirement Longitudinal Study (CHARLS) survey in 2015; a total of 2056 females and 2529 males were included in this study. According to the analysis findings, 46.15% of the respondents had depressive symptoms based on the CES-D, with a statistically significant gender difference of 54.32% in females and 39.50% in males. Logistic Regression findings identified that with regard to the items of physical housing environments, toilets without seats (OR = 1.349) and the unavailability of bathing facilities (OR = 1.469) were statistically associated with depressive symptoms among male participants, whereas for female participants the use of polluting fuels (OR = 1.248) and living arrangements (i.e., living with children, OR = 1.430) was statistically associated with depressive symptoms. Statistically significant gender differences were found for having shower or bath facilities and our findings underscored that physical housing environments and living arrangements were associated with depressive symptoms for both genders. Moreover, the study revealed that a slight gender difference exists in terms of geriatric depression in rural China. Females are more likely to become depressed than their male counterparts with the same characteristics.

## 1. Introduction

Ageing is one of the most conspicuous phenomena in the contemporary world. Between 2015 and 2050, the proportion of the world’s adults that is elderly is estimated to almost double from about 12% to 22% [1]. In absolute terms, there will be an expected increase from 900 million to 2 billion people over the age of 60 [2]. While ageing is a global phenomenon, it is progressing fastest in developing countries, especially in China. In 2016, the Chinese population aged 60 years and over was over 230 million, which will further increase to 480 million by 2050 [3].

Older adults face special physical and mental health challenges that need to be recognized. According to a World Health Organization report published in 2017, over 20% of older adults suffer from a mental or neurological disorder (excluding headache disorders), and 6.6% of all disability (disability-adjusted life years—DALYs) among people over 60 years was attributed to mental and neurological disorders. Depression, one of the most common mental disorders, affects approximately 7% of the world’s older population and comprises a substantial proportion of the global burden of diseases [4]. Research on depression in the elderly began in the 1980s and has emerged as a growing field since the 1990s.

The gender differences in the occurrence, development, and severity of geriatric depression have been well documented [5,6,7]. The higher prevalence of depression among female older adults has long been recognized. For example, previous studies suggested that female older adults are almost 1.3 to 2 times more likely than male older adults to meet the criteria for depressive symptoms [8,9]. However, the potential factors that lead to this gender difference vary from study to study and from population to population. Possible explanations of this gender difference in depression are complex social factors, such as social role and status, social support and participation, in addition to socioeconomic factors and health status [10,11]. Female older adults tend to have lower education levels and income than their male counterparts, and because of the difference in life expectancy they are more likely to lose their partner, which is an important risk factor for depressive symptoms.

Housing environment, as an important social determinant of health, has a well-documented association with depressive symptoms [12,13,14]. Housing environments contain physical, social, and psychological attributes. For instance, the physical environment may encompass tangible and observable attributes such as physical structure, design, housing facility, etc. [15,16,17]. The social and psychological factors encompass the living arrangement and the experience of community living such as neighborhood environments, forging relationships with neighbors, security, and belongingness. The association between housing environments and depressive symptoms has been investigated in several studies, particularly in urban areas [18,19]. Some previous studies have shown the relationship between urban housing environments and mental health [20]. However, research on housing environments and depression among the rural population is remarkably underdeveloped. Few studies have discussed in more detail the impact of rural housing environments on depressive symptoms among the elderly, especially in China [21]. In rural areas of China, poor physical housing environments may lead to a greater possibility of developing depressive symptoms. With ongoing urbanization, it is necessary to further focus on the relationship between rural housing environments and mental health, which has a significant effect on the construction of age-friendly environments and can further reduce the health inequality between the urban and rural population [22]. 

Living arrangements, an important component of social support that elderly people receive from their partners or family members, has commonly been viewed as a risk factor for depression in the elderly [23]. Previous research has shown that living arrangements are an independent risk factor contributing to depression [24,25]. Gender differences in the association between living arrangements and depression have also been demonstrated. Previous studies reported that older adults living alone were more depressed compared with those living with other family members [26,27]. Considering cultural factors, it has been suggested that the relationship between living arrangement and depression may be more prevalent in certain ethnic groups, especially among the Chinese population. 

We conducted this study to examine the gender differences in geriatric depressive symptoms in rural China, and further explored the influence of housing environments and living arrangements on depressive symptoms. We believe our study will improve the understanding of the complex nature of the gender differences in geriatric depressive symptoms, and the association between gender, housing environments, living arrangement, and depressive symptoms among the rural elderly population in China.

## 2. Materials and Methods

### 2.1. Design and Study Population

This study is based on the nationally representative China Health and Retirement Longitudinal Study (CHARLS) conducted by the China Centre for Economic Research of Peking University, which was designed after the Health and Retirement Study (HRS) in the USA as a broad purposed social science and health survey of the elderly in China. Face-to face interviews in respondents’ homes collected detailed information on their demographic characteristics, socioeconomic status, health-related behaviors and lifestyles, health status including health conditions, health insurance, and health services use [28]. More details on the CHARLS survey design are available from Zhao et al. Ethics approval for the baseline data collection was obtained from the Biomedical Ethics Review Committee of Peking University (IRB00001052-11015). Ethical approval for the use of CHARLS data was obtained from the University of Newcastle HREC (H-2015-0290). The data used for this study were from the third wave in 2015, and the inclusion criteria were: (1) age ≥ 60, (2) living in rural regions, (2) living at the survey site for at least six months, (3) being at home during the investigation period, and (4) able to participate in the study. Subjects with mental retardation and severe cognitive impairment had been excluded from the original research through a short screening form; only those respondents who needed little or no help with answering questions were allowed to advance. Thus, the number of respondents eligible for our analysis dropped to 4585 elderly.

### 2.2. Procedure

CHARLS utilized a multi-stage stratified probability-proportional-to-size sampling (PPS) technique to select participants. Face-to-face interviews were conducted to collect detailed information on demographic characteristics, socioeconomic status, health status including health conditions, health insurance, and health service use. The pilot survey was carried out in two provinces (Gansu and Zhejiang) in 2008, and was subsequently expanded to form the national baseline survey fielded in 2011. In the first wave 17,708 participants were surveyed; a total of 18,246 respondents aged 45 plus were followed up and surveyed in the second wave. In 2015, the number of participants increased to 21,095. CHARLS survey design has been described in more detail elsewhere [28,29].

### 2.3. Assessment and Measurements

#### 2.3.1. Depressive Symptoms

The 10-item Center for Epidemiologic Studies Depression Scale (CES-D) was used to screen depressive symptoms. The time frame for the CES-D questions referred to the week prior to the interview. Each item was rated on a 4-point Likert scale, with answers varying from ‘rarely or none of the time (0–1 day)’ to ‘most or all of the time (5–7 days)’. The total scores ranged from 0 to 30, with a lower score indicating a lower level of depressive symptoms. CES-D has shown good validity and reliability in the Chinese population. A previous validation study among older adults found that a cutoff point of 10 provides the optimal threshold to identify clinically significant depressive symptoms. Thus, a cutoff point of 10 was used in this study to generate the dichotomous depressive symptoms variable. Participants who scored lower than 11 were classified as non-depressed. The CESD-10 has been described in detail elsewhere [29].

#### 2.3.2. Housing Environment

Two aspects of housing environment were assessed: the physical environment and the living arrangements.

##### Physical Environment

Five variables were used to measure physical environment: housing materials were dichotomized as ‘improved material’ (concrete and steel/bricks and wood) and ‘unimproved material’ (adobe, wood, cave dwelling, Mongolian yurt/woolen felt, stone); Cooking fuel was categorized as Clean fuel (2 = natural gas, 3 = marsh gas, 4 = liquefied petroleum gas, 5 = electric), Polluting fuel (1 = coal, 6 = crop residue/wood burning), and others (7 = other). Housing facility was assessed by the availability of a toilet, running water, and a shower or bath facility. These facilities were all categorized as “0 = yes” (available) and “1 = no” (not available), while toilets were categorized as “0 = toilet with seat” or “1 = toilet without seat.”

##### Living Arrangements

The instrument of living arrangement contained three items: living with spouse (yes or no), living with children (yes or no), and living alone (yes or no).

#### 2.3.3. Health Status

In this study, health status was assessed by “number of chronic diseases,” “disability status,” and “activities of daily living (ADL) limitations.”

##### Number of Non-Communicable Diseases (NCD_S_)

NCD_S_ was measured as the cumulative number of diagnosed chronic conditions by a physician (hypertension, dyslipidemia, diabetes, cancer, chronic lung diseases, liver or gallbladder disease, heart disease, stroke, kidney disease, stomach or other digestive disease, emotional, nervous, or psychiatric problems, memory-related disease, rheumatism/arthritis, asthma) on three scales: 0, 1–2, ≥3 [22].

##### Disability Status

Disability status was measured by asking participants: “Do you have one of the following disabilities?” Responses were categorized as “yes” when they had any disability of physical disabilities, brain damage/mental retardation, vision problem, hearing problem, or speech impediment.

##### ADL

ADL limitations indicate any self-reported difficulty in any of the following six activities of daily living: eating, dressing, getting into or out of bed, using the toilet, bathing/showering, or controlling urination and defecation. The four response options were: 1 = “I don’t have any difficulty,” 2 = “I have difficulty but can still do it,” 3 = “I have difficulty and need help,” 4 = “I cannot do it.” It was dichotomized as “unimpaired” vs. “impaired.” “ADL-impaired” was defined as “have difficulty and need help” or “cannot do it” in any ADL item.

#### 2.3.4. SES

Educational level and personal annual income were used to determine SES. Education levels were classified as “Primary school and below” or “High school and above.” Personal annual income was categorized to “≤1477 dollars,” “1477–2954 dollars,” or “≥2954 dollars.”

#### 2.3.5. Socio-Demographic Characteristics

Socio-demographic variables included age (year), gender (female, male), and marital status (married, unmarried).

### 2.4. Statistical Analysis

All statistical analyses and tests were conducted using Stata version 13 (Stata, College Station, TX, USA) [28]. Data are presented with percentages and proportions for categorical values. First, we conducted a χ^2^ test to assess the statistical differences between groups. Then, we conducted a binary logistic regression to assess the associations of depressive symptoms. Finally, we performed a *t*-test analysis to assess the interaction effects of gender on these associations. In all analyses, the criterion for statistical significance was *p* < 0.05.

## 3. Results

Table 1 reports descriptive statistics for the participants by genders. Out of 4585 rural participants 44.84% are females and 55.16% are males. Most respondents had lower level of education (elementary school and below), with significantly more men in the low-level group. Approximately 46.15% of the respondents had depressive symptoms based on the CES-D, with a statistically significant gender difference of 54.32% in females and 39.50% in males.

Table 1 also shows depressive symptoms for both female and male participants were significantly associated with age, annual income, number of NCDS, disabilities status, and ADL limitations. The depressed participants tended to be older and have a lower personal income for both genders. Likewise, participants with depressive symptoms reported more chronic diseases, a greater proportion of physical disability, and ADL limitations. Among female older adults, the depressed were more often unmarried, whereas the depressed male participants tended to have a lower education level than those who were not reportedly depressed (Table 1).

Table 2 shows the prevalence of depressive symptoms by genders according to physical housing environments and living arrangements. Depressive symptoms for both genders were only significantly associated with three items of physical housing environments (the type of cooking fuel and toilet; the availability of a shower or bath). In addition, depressive symptoms were significantly associated with living arrangements among female participants, whereas depressed male participants tended to have a lower education level than those who were not reportedly depressed.

The final logistical regression results are shown in Table 3. Regression findings suggest lower personal income (≤10000: OR = 2.448, female; OR = 1.432, male), disability (OR = 1.318, female; OR = 1.786, male), ADL dysfunction (OR = 1.539, female; OR = 1.297, male), and more than two types of chronic diseases (OR = 2.448, female; OR = 1.432, male) are significantly and positively associated with geriatric depressive symptoms for both female and male participants. With regard to physical housing environments, a toilet without a seat (OR = 1.349) and a lack of bathing facilities (OR = 1.469) are statistically associated with depressive symptoms among female participants, whereas depressed male participants tend to use polluting fuel (OR = 1.248). Remarkably, the living arrangement (living with children, OR = 1.430) is only statistically associated with depressive symptoms among female participants.

Statistically significant gender differences in these associations were found for having a physical disability and no shower or bath. Test statistics of the interactions for gender are shown in Table 4.

## 4. Discussion

### 4.1. Key Findings

Our study examined the gender differences in geriatric depressive symptoms in rural China, and further explored the influence of housing environments and living arrangements on depressive symptoms. In this study, a high prevalence of depressive symptoms was found in both genders, with a preponderance in females, which is in line with previous studies. Females were found to be more often depressed than males. Around 46.15% of the respondents had depressive symptoms according to the CES-D, with a statistically significant gender difference of 54.32% in men and 39.50% in women. Previous epidemiologic studies suggested that female older adults are 1.3–2 times more likely than male older adults to meet the screening criteria for depressive symptoms [8,10]. Numerous demographical, biological, social and psychological explanations for these gender differences have been proposed. Psychological factors include personality, coping styles and cognitive abilities. Previous studies reported that males report fewer psychological symptoms leading to difficulties in detecting depression. Females are also approximately twice as likely as males to be diagnosed with generalized anxiety disorder [11,30] and score more highly on self-reported scales measuring anxiety [31].

In our study, using a polluting cooking fuel is statistically associated with depressive symptoms among male participants. Indoor air pollution (IAP), one of the major public health concerns in low- and middle-income countries, is mainly caused by the use of polluting cooking fuels such as coal, charcoal, crop residue, and wood burning. According to a WHO report in 2016, nearly 3 billion (40%) of the world’s people rely on solid fuels, including coal and biomass, for domestic cooking [32,33,34,35]. Evidence shows that IAP is associated with poor physical health. Prior researchers have identified IAP as the most important environmental risk factor globally associated with adverse health effects [36,37,38,39,40,41,42,43,44,45,46,47,48]. However, there is little empirical evidence of the relationship between IAP and depression based on nationally representative data, especially in developing countries [39,40]. In general, the negative effects of polluting cooking fuel on depressive symptoms have not been given enough attention because they are largely dependent on the population group [41,42].

We found that using a toilet without a seat is statistically associated with depressive symptoms among female participants. However, very little research has explored the relationship between toilet type and depressive symptoms. Research on health, especially on microbiological pollution, has demonstrated that the species of microbes on squat pans are roughly the same as on toilets, but the number alone is much higher [43]. Some other previous investigations have suggested that the use of squat pans can over time cause anal fissure to a certain extent, raise the risk of dizziness, and even lead to falls [44,45]. Perhaps we can deduce from the existing research that the existence of chronic diseases resulting from using a toilet without a seat may lead to a greater possibility of depression among the elderly.

In our study, we found that having no bathing facility is statistically associated with depressive symptoms among female participants. In terms of the relationship between shower/bath facilities and depressive symptoms, we found only a few articles, in the literature study, that involved the relationship between bath facilities and health outcome. A randomized clinical pilot trial conducted in Germany showed that hypothermic baths (HTB) do have generalized efficacy in depressed patients. The results of a non-controlled HTB study aimed at 20 depressive patients, also conducted in Germany, showed an improvement after hypothermic baths [46,47]. Furthermore, HTB, especially before bedtime, improved sleep quality in healthy, insomniac people and elderly patients with depression and vascular dementia. Whole-body hyperthermia, according to a further non-controlled study, showed a significant reduction in CES-D among 16 depressive patients. Our research further corroborated the previous research. The prior research suggests that HTB have antidepressant effects that are mediated through changes in circadian functioning and temperature physiology, although the underlying mechanisms remain unclear [48].

Furthermore, the type of toilet and cooking fuel, and access to bathing facilities, as important symptoms of poverty, have associations with the process of developing depressive symptoms and thus reflect the impact of socioeconomic status (SES) on an individual’s mental health. It has been suggested that poverty/poor SES may lead to poor access to mental health services, and further affect the diagnosis and treatment of depression, as it is very difficult for low-income populations to meet regular health care needs and be screened for depressive symptoms [49,50,51,52]. How to approach these problems is outside the scope of this paper, but our findings highlight the importance of poverty or poor SES in the development of depressive symptoms in rural China.

In the face of psychosocial housing environments, living with children was significantly associated with depression only in females, although these gender differences were not statistically significant. This is consistent with previous studies that social support from children might be recognized as more valuable than from other family members, especially for females [10,25]. Living arrangement, an important component of the social support that elderly people receive from family members, has commonly been viewed as a risk factor for depression in the elderly. Adequate social support and participation are generally regarded as an important protective factor against depressive symptoms in the elderly [50]. We surmise that the relationship between traditional culture and mental health may be more prevalent among the Chinese population. Chinese proverbs include “Raise children to provide against old age” and Confucius said, “While one’s parents are alive, one should not go far away from his or her parents.” This may explain the protective effect on older adults in the context of Chinese culture. One important explanation might be that adult children are important sources of psychological and financial support to elderly adults in China [17]. Previous research showed that more than 60% of older adults’ money comes from their children [51,52]. Moreover, the concept of filial piety from Chinese traditional culture is still critical to the social support system of modern Chinese families: children are still the main source of social support to their older parents. Not surprisingly, then, living arrangement might have a greater impact on mental health in aging Chinese population due to the cultural ideal of social support in later life.

### 4.2. Strengths and Limitations

Our study adds to the existing knowledge highlighting the role of physical and psychological housing environments in exploring the development of depressive symptoms in rural China. Furthermore, the use of nationally representative data from all of China contributes to the implementation of cross-country comparisons and to the collection of much more information about the effect of physical housing environments and living arrangements on depressive symptoms. The main limitations are a cross-sectional study design and the use of self-reported data. Caution must be taken when generalizing the findings of this study to the rest of the population in China since it is a limited sample. There was no further clinical diagnosis or treatment for the participants who met the screening criteria according to CES-D, in the original research.

### 4.3. Recommendations

Our findings underscore the importance of strong political commitment, inter-sectorial coordination, and adequate financing in order to prevent the development of depressive symptoms among the elderly in rural China. Overall, our findings reveal that more attention should be paid to the relationship between gender, housing environment, living arrangement, and depressive symptoms among the rural elderly population in developing countries.

## 5. Conclusions

Statistically significant gender differences were found for having shower or bath facilities and our findings underscored that physical housing environments and living arrangements were associated with depressive symptoms for both genders. Moreover, the study revealed that a slight gender difference exists in terms of geriatric depression in rural China. Females are more likely to become depressed than their male counterparts with the same characteristics.

## Figures and Tables

**Table 1:** Distribution and prevalence of depressive symptoms by gender according to participant characteristics.

Variable	Full Samples		*p*	Female		*p*	Male		*p*
	Not Depressed (*n* = 2469)	Depressed (*n* = 2116)		Not Depressed (*n* = 939)	Depressed (*n* = 1117)		Not Depressed (*n* = 1530)	Depressed (*n* = 999)	
**Age (years)**			<0.001			<0.001			0.028
60–74	1908(77.28)	1488(70.32)		692(73.70)	731(65.44)		1216(79.48)	757(75.78)	
≥75	561(22.72)	628(29.68)		247(26.30)	386(34.56)		314(20.52)	242(24.22)	
Marital status			<0.001			<0.001			0.081
Married	1735(70.27)	1273(60.16)		507(53.99)	500(44.76)		1228(80.26)	773(77.38)	
Unmarried	734(29.73)	843(39.84)		432(46.01)	617(55.24)		302(19.74)	226(22.62)	
Education			0.002			0.576			0.026
Elementary school and below	2309(93.52)	2024(95.65)		909(96.81)	1086(97.22)		1400(91.50)	938(93.89)	
Middle school and above	160(6.48)	92(4.35)		30(3.19)	31(2.78)		130(8.50)	61(6.11)	
Personal annual income (dollar)			<0.001			<0.001			<0.001
≤1477	1871(75.78)	1772(83.74)		747(79.55)	951(85.14)		1124(73.46)	821(82.18)	
1477–2954	334(13.53)	222(10.49)		110(11.71)	124(11.10)		224(14.64)	98(9.81)	
≥2954	264(10.69)	122(5.77)		82(8.74)	42(3.76)		182(11.90)	80(8.01)	
Number of NCDs			<0.001			<0.001			<0.001
0	458(18.55)	240(11.34)		156(16.61)	109(9.76)		302(19.74)	131(13.11)	
1–2	1396(56.54)	1219(57.61)		525(55.91)	656(58.73)		871(56.93)	563(56.36)	
≥3	615(24.91)	657(31.05)		258(27.48)	352(31.51)		357(23.33)	305(30.53)	
Disability			<0.001			<0.001			<0.001
Yes	999(40.46)	1176(55.58)		411(43.77)	615(55.06)		588(38.43)	561(56.16)	
No	1479(59.54)	940(44.42)		528(56.23)	502(44.94)		942(61.57)	438(43.84)	
ADL			<0.001			<0.001			0.001
Unimpaired	2133(86.39)	1695(80.10)		826(87.97)	892(79.86)		1307(85.42)	803(80.38)	
Impaired	336(13.61)	421(19.90)		113(12.03)	225(20.14)		223(14.58)	196(19.62)	

**Table 2:** Distribution and prevalence of depressive symptoms by gender according to physical housing environments and living arrangements.

Variable	Full Samples		*p*	Female		*p*	Male		*p*
	Not Depressed (*n* = 2469)	Depressed (*n* = 2116)		Not Depressed (*n* = 939)	Depressed (*n* = 1117)		Not Depressed (*n* = 1530)	Depressed (*n* = 999)	
**Physical environments**									
Housing materials			0.008			0.054			0.051
Improved	1958(79.30)	1609(76.04)		751(79.98)	854(76.45)		1207(78.88)	755(75.58)	
Unimproved	511(20.70)	628(23.96)		188(20.02)	263(23.55)		323(21.12)	244(24.42)	
Cooking fuel			<0.001			0.009			<0.001
Clean fuel	1156 (46.82)	837(39.56)		436(46.43)	445(39.84)		720(47.06)	392(39.24)	
Polluting fuel	1287(52.12)	1257(59.40)		497(52.93)	661(59.18)		790(51.63)	596(59.66)	
others	26(1.06)	22(1.04)		6(0.64)	11(0.98)		20(1.31)	11(1.10)	
Type of toilet			<0.001			0.001			0.023
Toilet with seat	370(15.06)	233(11.12)		149(15.87)	120(10.74)		221(14.44)	113(11.31)	
Toilet without seat	2087(84.94)	1862(88.88)		790(84.13)	997(89.26)		1309(86.56)	886(85.09)	
Running water			0.005			0.063			0.008
Yes	1729(70.03)	1399(66.11)		676(71.99)	762(68.22)		1053(68.82)	637(63.76)	
No	740(29.97)	717(33.89)		263(28.01)	355(31.78)		477(31.18)	362(36.24)	
Shower or bath facility			<0.001			<0.001			0.004
Yes	1128(45.69)	788(37.24)		435(46.33)	394(35.27)		693(45.29)	394(39.44)	
No	1341(54.31)	1328(62.76)		504(53.67)	723(64.73)		837(54.71)	605(60.56)	
**Living arrangement**									
Living with spouse (Yes)	1634(66.18)	1195(56.47)	<0.001	473(50.37)	463(41.45)	<0.001	1161(75.88)	732(73.27)	0.139
Living with children (Yes)	1073(43.46)	1034(48.87)	<0.001	409(43.56)	565(50.58)	0.001	664(43.40)	469(46.95)	0.079
Living alone (Yes)	461(18.67)	449(21.22)	0.031	236(25.13)	298(26.68)	0.426	225(14.71)	151(15.12	0.777

**Table 3:** Logistics regression of physical housing environments and living arrangements associated with depressive symptoms by gender.

Variable	Categories	Model 1, Female			Model 2, Male		
		0R	95%CI	*p*	0R	95%CI	*p*
Age (ref. 60–74)	≥75	1.354	1.098–1.670	0.005	1.072	0.874–1.315	0.505
Marital status (ref. Married)	Unmarried	0.998	0.608–1.637	0.993	1.019	0.664–1.565	0.930
Education (ref. Elementary school and below)	Middle school and above	1.033	0.606–1.759	0.906	0.878	0.632–1.218	0.435
Personal annual income (ref. ≥2954)	1477–2954	2.285	1.428–3.655	0.001	0.940	0.654–1.351	0.738
	≤1477	2.448	1.635–3.666	<0.001	1.432	1.068–1.920	0.017
Number of NCDs (ref. 0)	1–2	1.597	1.206–2.115	0.001	1.313	1.034–1.667	0.025
	≥3	1.791	1.402–2.578	<0.001	1.665	1.276–2.172	<0.001
Disability (ref. No)	Yes	1.318	1.094–1.587	0.004	1.786	1.507–2.117	<0.001
ADL (ref. Unimpaired)	Impaired	1.539	1.193–1.985	0.001	1.297	1.042–1.616	0.020
Cooking fuel (ref. Clean fuel)	Polluting fuel	1.066	0.879–1.292	0.517	1.248	1.045–1.490	0.015
	others	1.530	0.538–4.350	0.425	0.965	0.448–2.076	0.927
Type of toilet (ref. Toilet with seat)	Toilet without seat	1.349	1.023–1.778	0.034	1.193	0.923–1.542	0.177
Running water (ref. Yes)	No	1.053	0.858–1.292	0.622	1.132	0.946–1.354	0.176
Shower or bath facility (ref. Yes)	No	1.469	1.202–1.796	<0.001	1.036	0.862–1.245	0.704
Living with spouse (ref. No)	Yes	0.858	0.525–1.403	0.542	1.004	0.673–1.498	0.986
Living with children (ref. Yes)	No	1.430	1.186–1.726	<0.001	1.179	0.996–1.395	0.055

**Table 4:** Regression coefficients across models: interaction effects.

Variable	Female vs. Male	
	χ^2^t	*p*
Age	2.43	0.119
Marital status	0.00	0.968
Education	0.33	0.568
Personal annual income	0.93	0.335
Number of NCDs	0.17	0.676
Disability	5.98	0.014
ADL	1.30	0.255
Cooking fuel	0.67	0.412
Type of toilet	0.66	0.416
Running water	0.36	0.548
Shower or bath facility	6.20	0.013
Living with spouse	0.15	0.696
Living with children	2.04	0.153

## References

[1] World Report on Ageing and Health 2015. http://www.who.int/ageing/events/world-report-2015-launch/en/.

[2] UNFPA and HelpAge International (2012). Ageing in the Twenty-First Century: A Celebration and a Challenge. https://www.unfpa.org/publications/ageing-twenty-first-century.

[3] UNFPA and HelpAge International Ageing. https://www.unfpa.org/ageing.

[4] Mental Health of Older Adults. http://www.who.int/mediacentre/factsheets/fs381/en/.

[5] Kessler R.C., McGonagle K.A., Swartz M., Blazer D.G., Nelson C.B. (1993). Sex and depression in the National Comorbidity Survey: I. Lifetime prevalence, chronicity and recurrence. J. Affect. Disord..

[6] Pachana N.A., McLaughlin D., Leung J., Byrne G., Dobson A. (2012). Anxiety and depression in adults in their eighties: Do gender differences remain?. Int. Psychogeriatr..

[7] Sonnenberg C.M., Deeg D.J.H., van Tilburg T.G., Vink D., Beekman A.T.F. (2013). Gender differences in the relation between depression and social support in later life. Int. Psychogeriatr..

[8] Jorm A.F. (1987). Sex and age differences in depression: A quantitative synthesis of published research. Aust. N. Z. J. Psychiatry.

[9] Silverstein B., Edwards T., Gamma A., Ajdacic-Gross V., Rossler W., Angst J. (2013). The role played by depression associated with somatic symptomatology in accounting for the gender difference in the prevalence of depression. Soc. Psychiatry Psychiatr. Epidemiol..

[10] Schuch J.J., Roest A.M., Nolen W.A., Penninx B.W., de Jonge P. (2014). Gender differences in major depressive disorder: Results from the Netherlands study of depression and anxiety. J. Affect. Disord..

[11] Kim J.H., Cho M.J., Hong J.P., Bae J.N., Cho S.J., Hahm B.J., Lee D.W., Park J.I., Lee J.Y., Jeon H.J., Chang S.M. (2015). Gender differences in depressive symptom profile: Results from nationwide general population surveys in Korea. J. Korean Med. Sci..

[12] Wright P.A., Kloos B. (2006). Housing environment and mental health outcomes: A levels of analysis perspective. J. Environ. Psychol..

[13] Alkhatib I.A., Arafat R.N., Musmar M. (2005). Housing environment and women’s health in a Palestinian refugee camp. J. Int. J. Environ. Health Res..

[14] Pappa E., Chatzikonstantinidou S., Chalkiopoulos G., Papadopoulos A., Niakas D. (2015). Health-Related Quality of Life of the Roma in Greece: The Role of Socio-Economic Characteristics and Housing Conditions. Int. J. Environ. Res. Public Health.

[15] Lawrence R.J. (2017). Constancy and Change: Key Issues in Housing and Health Research, 1987–2017. Int. J. Environ. Res. Public Health.

[16] Butterfield M.C., Williams A.R., Beebe T., Finnie D., Liu H., Liesinger J., Sloan J., Wheeler P.H., Yawn B., Juhn Y.J. (2011). A two-county comparison of the HOUSES index on predicting self-rated health. J. Epidemiol. Community Health.

[17] Nguyen Q.C., Rehkopf D.H., Schmidt N.M., Osypuk T.L. (2016). Heterogeneous Effects of Housing Vouchers on the Mental Health of US Adolescents. Am. J. Public Health.

[18] Christensen J. (2016). Indigenous housing and health in the Canadian North: Revisiting cultural safety. J. Health Place.

[19] Gibson M., Petticrew M., Bambra C., Sowden A.J., Wright K.E., Whitehead M. (2011). Housing and health inequalities: A synthesis of systematic reviews of interventions aimed at different pathways linking housing and health. Health Place.

[20] Thomson H., Thomas S., Sellstrom E., Petticrew M. (2009). The health impacts of housing improvement: A systematic review of intervention studies from 1887 to 2007. Am. J. Public Health.

[21] Suglia S.F., Duarte C.S., Sandel M.T. (2011). Housing Quality, Housing Instability, and Maternal Mental Health. J. Urban Health Bull. N. Y. Acad. Med..

[22] Byck G.R., Bolland J., Dick D., Swann G., Henry D., Mustanski B. (2015). Effect of housing relocation and neighborhood environment on adolescent mental and behavioral health. J. Child Psychol. Psychiatry.

[23] Alexander B.B., Rubinstein R.L., Goodman M., Luborsky M. (1992). A path not taken: A cultural analysis of regrets and childlessness in the lives of older women. Gerontologist.

[24] Chou K.L., Chi I. (2000). Comparison between elderly Chinese living alone and those living with others. J. Gerontol. Soc. Work.

[25] Chou K.L., Ho A.H.Y., Chi I. (2006). Living alone and depression in Chinese older adults. Aging Ment. Health.

[26] Beekman A.T., Deeg D.J., Smit J.H., van Tilburg W. (1995). Predicting the course of depression in the older population: Results from a community-based study in The Netherlands. J. Affect. Disord..

[27] Ng K.M., Lee T.M.C., Chi I. (2004). Relationship between living arrangements and the psychological well-being of older people in Hong Kong. Australas. J. Ageing.

[28] Li L.W., Liu J., Zhang Z., Xu H. (2015). Late-life Depression in Rural China: Do Village Infrastructure and Availability of Community Resources Matter. Int. J. Geriatr. Psychiatry.

[29] Cheng H.G., Chen S., McBride O., Phillips M.R. (2016). Prospective relationship of depressive symptoms, drinking, and tobacco smoking among middle-aged and elderly community-dwelling adults: Results from the China Health and Retirement Longitudinal Study (CHARLS). J. Affect. Disord..

[30] Nolen-Hoeksema S. (2001). Gender differences in depression. Curr. Dir. Psychol. Sci..

[31] Leach L.S., Christensen H., Mackinnon A.J., Windsor T.D., Butterworth P. (2008). Gender differences in depression and anxiety across the adult lifespan: The role of psychosocial mediators. Soc. Psychiatry Psychiatr. Epidemiol..

[32] Naz S., Page A., Agho K.E. (2017). Household air pollution from use of cooking fuel and under-five mortality: The role of breastfeeding status and kitchen location in Pakistan. PLoS ONE.

[33] Hou B.D., Tang X., Ma C., Liu L., Wei Y.M., Liao H. (2016). Cooking fuel choice in rural China: Results from microdata. J. Clean. Prod..

[34] Owili P.O., Muga M.A., Pan W.C., Kuo H.W. (2017). Cooking fuel and risk of under-five mortality in 23 Sub-Saharan African countries: A population-based study. Int. J. Environ. Health Res..

[35] Xiaokang L.V., Cong W.A.N.G. (2017). Exposure to Air pollution impairs cognitive function and psychological well-being. Adva. Psychol. Sci..

[36] Asikainen A., Carrer P., Kephalopoulos S., de Oliveira Fernandes E., Wargocki P., Hänninen O. (2016). Reducing burden of disease from residential indoor air exposures in Europe (HEALTHVENT project). Environ. Health.

[37] Pollard S.L., D’Ann L.W., Breysse P.N., Baron P.A., Grajeda L.M., Gilman R.H., Miranda J.J., Checkley W. (2014). A cross-sectional study of determinants of indoor environmental exposures in households with and without chronic exposure to biomass fuel smoke. J. Environ. Health.

[38] Painschab M.S., Davila-Roman V.G., Gilman R.H., Vasquez-Villar A.D., Pollard S.L., Wise R.A., Miranda J.J., Checkley W., CRONICAS Cohort Study Group (2013). Chronic exposure to biomass fuel is associated with increased carotid artery intima-media thickness and a higher prevalence of atherosclerotic plaque. Heart.

[39] Fatmi Z., Coggon D. (2016). Coronary heart disease and household air pollution from use of solid fuel: A systematic review. J. Br. Med. Bull..

[40] Sehgal M., Rizwan S.A., Krishnan A. (2014). Disease burden due to biomass cooking-fuel-related household air pollution among women in India. Glob. Health Action.

[41] Peng N., Alfonso S P., Xue J. (2016). Fuel for Life: Domestic Cooking Fuels and Women’s Health in Rural China. Int. J. Environ. Res. Public Health.

[42] Khan M.N., Islam M.M., Islam M.R., Rahman M.M. (2017). Household air pollution from cooking and risk of adverse health and birth outcomes in Bangladesh: A nationwide population-based study. Environ. Health.

[43] Li C., Liang Y., Yang Y., Li Y., Xing W. (2017). Evaluation of squat pan and toilet in sanitation, health and water-saving. J. Inner Mong. Environ. Sci..

[44] Mahdavinejad M., Bemanian M., Farahani S.F., Tajik A. (2011). Role of toilet type in transmission of infections. Acad. Res. Int..

[45] Erel S., Adahan D., Kismet K., Caylan A., Tanrikulu Y., Akkus M.A. (2009). Risk factors special to eastern culture for the development of anal fissure. Bratislavské Lekárske Listy.

[46] Ono J., Hashiguchi N., Sawatari H., Ohkusa T., Miyazono M., Son S.Y., Magota C., Tochihara Y., Chishaki A. (2017). Effect of water bath temperature on physiological parameters and subjective sensation in older people. Geriatr. Gerontol. Int..

[47] Horne J.A., Reid A.J. (1985). Night-time sleep EEG changes following body heating in a warm bath. Electroencephalogr. Clin. Neurophysiol..

[48] Naumann J., Grebe J., Kaifel S., Weinert T., Sadaghiani C., Huber R. (2017). Effects of hyperthermic baths on depression, sleep and heart rate variability in patients with depressive disorder: A randomized clinical pilot trial. BMC Complement. Altern. Med..

[49] Gallo L.C., Matthews K.A. (2003). Understanding the association between socioeconomic status and physical health: Do negative emotions play a role?. Psychol. Bull..

[50] Vonneilich N., Jöckel K.H., Erbel R., Klein J., Dragano N., Siegrist J., von Dem Knesebeck O. (2012). The mediating effect of social relationships on the association between socioeconomic status and subjective health—Results from the Heinz Nixdorf Recall cohort study. BMC Public Health.

[51] Jeon G.S., Choi K., Cho S.I. (2017). Impact of Living Alone on Depressive Symptoms in Older Korean Widows. Int. J. Environ. Res. Public Health.

[52] Dalgard O.S., Dowrick C., Lehtinen V., Vazquez-Barquero J.L., Casey P., Wilkinson G., Ayuso-Mateos J.L., Page H., Dunn G., ODIN Group (2006). Negative life events, social support and gender difference in depression: A multinational community survey with data from the ODIN study. Soc. Psychiatry Psychiatr. Epidemiol..

